# The impact of clinical symptoms and endoscopic and histologic disease activity on health-related quality of life in patients with ulcerative colitis following treatment with multimatrix mesalazine

**DOI:** 10.1007/s11136-021-02787-4

**Published:** 2021-03-02

**Authors:** Aaron Yarlas, Mary Kaye Willian, Arpita Nag

**Affiliations:** 1 QualityMetric, 1301 Atwood Avenue, Suite 216E, Johnston, RI 02919 USA; 2 Shire, 725 Chesterbrook Boulevard, Wayne, PA 19087 USA; 3 Shire, 300 Shire Way, Lexington, MA 02421 USA; 4 Present Address: Sanofi, 270 Albany St, Cambridge, MA 02139 USA

**Keywords:** Ulcerative colitis, Quality of life, Histology, Endoscopy, Clinical symptoms

## Abstract

**Purpose:**

Studies of patients with ulcerative colitis (UC) report that reduced clinical symptoms and endoscopic activity predict better health-related quality of life (HRQoL). However, no study has examined the joint and unique associations of clinical and endoscopic activity with HRQoL, nor of histologic inflammation and HRQoL. These post hoc analyses evaluated whether reduced clinical, endoscopic, and histologic disease activity were uniquely associated with improved HRQoL for adults with active mild-to-moderate UC receiving once-daily 4.8 g/day multimatrix mesalazine for 8 weeks.

**Methods:**

Assessments at baseline and week 8 (i.e., treatment completion) included clinical and endoscopic activity (modified UC-Disease Activity Index), histology (Geboes scoring), and HRQoL (Short Inflammatory Bowel Disease Questionnaire [SIBDQ]; SF-12v2^®^ Health Survey [SF-12v2]). Associations among each type of disease activity and HRQoL were examined by correlations and by mean changes in SIBDQ and SF-12v2 scores between disease activity subgroups (e.g., achievement of clinical remission; mucosal healing). Regression models estimated unique variance in HRQoL accounted by each type of disease activity.

**Results:**

Within the analysis sample (*n* = 717), patients with reduced clinical and endoscopic activity had significantly larger improvements in all HRQoL domains (*p* < 0.001), as did patients in both endoscopic and clinical remission compared to patients in endoscopic remission only (*p* < 0.05). Patients with histologic activity post-treatment scored significantly worse on all HRQoL domains than patients with no activity (*p* < 0.05). Correlations and regression models found that decreases in clinical and endoscopic activity were associated with improvements in HRQoL domain scores.

**Conclusions:**

Clinical symptoms and mucosal health have separable, distinct impacts on UC patients’ HRQoL.

**Supplementary Information:**

The online version of this article (10.1007/s11136-021-02787-4) contains supplementary material, which is available to authorized users.

## Introduction

Ulcerative colitis (UC) is a chronic, recurrent inflammatory bowel disease (IBD) characterized by inflammation of the colon and rectum. Health-related quality of life (HRQoL) is a multidimensional concept that captures impacts of a health condition, and its treatment, on an individual’s daily physical, emotional, mental, and social functioning, as well as the impact of the individual’s perceived health on their ability to live a fulfilling life [1, 2]. In recent years, HRQoL has been recognized as an important outcome when evaluating health, and improvements in health due to treatment and quality of care, in patients with UC [3]. When in remission, patients with UC are typically asymptomatic, with HRQoL similar to the general population [4–7]. However, patients with active UC often experience fatigue, increased need to defecate, diarrhea, bloody stools, and abdominal pain. HRQoL is typically sub-normal in patients experiencing acute flares [5, 8] while effective treatment of patients with active UC has been shown to improve HRQoL [9–13].

The effectiveness of treatment for patients with active UC in clinical trials is typically evaluated by the likelihood of inducing clinical remission and mucosal healing. Clinical remission is indicated as a substantial reduction of clinical symptoms, while mucosal healing reflects a sizable decrease in inflammation and ulceration of colonic and rectal tissue, as evidenced from endoscopic and histologic assessments [14]. In clinical practice, evidence for patients’ treatment response has typically focused on clinical symptoms. Following evidence showing that inflammation in the mucosa is associated with an increased likelihood of relapse [15–18] as well as increased risk of developing colorectal cancer [19–21] and colectomy [22, 23], there has recently been more emphasis on mucosal healing as a goal of UC therapy, accompanied by calls for including endoscopic and histologic endpoints in clinical trials [24–29].

While previous studies have established that increased HRQoL for patients with UC is predicted by reduced clinical symptoms [30–32] as well as improved mucosal health [33–37], none of these studies assessed whether improvements in clinical symptoms and mucosal healing are uniquely associated with improvements in HRQoL. Findings regarding the independence of clinical symptoms and mucosal health are mixed: some studies report very strong correlations between the two [38–40], but others report marked dissociations, with numerous patients in clinical remission who do not exhibit mucosal healing, and vice versa [41–43]. Given these inconsistencies, it is unclear whether changes in HRQoL are uniquely associated with changes in both clinical symptoms and mucosal health. The current study examines whether changes in clinical, endoscopic, and histologic disease activity are uniquely associated with meaningful changes in HRQoL for patients with UC following disease treatment.

## Methods

### Study design and sample

Data in the current post hoc analyses were from the MOMENTUM trial (ClinicalTrials.gov Identifier: NCT01124149), a phase 3b/4 open-label, multinational, single-arm prospective study of adults with mild-to-moderate UC treated with multimatrix mesalazine. Key exclusion criteria for this trial included severe UC; diagnosis of Crohn’s disease or proctitis; positive stool culture for enteric pathogens; previous colonic surgery; moderate or severe renal and/or hepatic impairment; systemic or rectal steroid use within the 4 weeks prior to screening; and history of biologic (anti–tumor necrosis factor agent) use. The study consisted of an initial induction phase followed by a maintenance phase. During the induction phase, patients with active UC received 4.8 g/day of multimatrix mesalazine once daily (QD) for up to 8 weeks. Analyses reported here include data from only the induction phase. Outcomes measures during the induction phase were assessed at the pretreatment screening or baseline visit, and at patients’ final visit (the week 8 visit for treatment completers or the early withdrawal [EW] visit for non-completers). A more detailed description of the sample and study design for the MOMENTUM trial has been published elsewhere [44].

This study was approved by Institutional Review Boards at each study site (see Table 1 in the Electronic Supplementary Material). Only patients who provided written informed consent at screening were eligible for enrollment in this study.

### Assessments

#### Clinical symptoms and endoscopy

Clinical and endoscopic disease activity were measured using a modified version^1^ of the UC-Disease Activity Index (UC-DAI) [45], which consists of 4 items: 2 patient reported (stool frequency and rectal bleeding) and two physician reported (mucosal appearance and physician’s global assessment). All items include 4 response options (0–3; see Table 2 in the ESM for coding of scores), with higher scores indicating more disease activity. A total score (range 0–12) can be calculated as the sum of each item score. Assessment of patient-rated UC-DAI items at the end of treatment was based on the average of scores recorded by patients for the last available 3 days within the 5-day period immediately prior to the week 8/EW visit. Scores for patient-rated items were reported by patients using an Interactive Voice Response System (IVRS). Scores for clinician-rated items were reported by clinicians using either IVRS or electronically via an Interactive Web Response System (IWRS).

#### Histology

At screening or baseline, histologic assessment was based on examination of either 2 or 4 biopsies. For each patient, 2 biopsies were taken from the rectum. When the rectum was not the area with the highest score of inflammation, two additional biopsies were taken from the area where inflammation scored the highest. Biopsies taken at the week 8/EW visit were from the same area(s) as the biopsies taken at the screening/baseline visit.

Histologic activity for each biopsied area was graded by a histopathologist (who was blinded to patients’ clinical course and endoscopic findings) using a modified version^2^ of Geboes scoring system [46] (see Table 3 in the ESM). Patients’ maximum histologic score was analyzed as a categorical outcome: endpoints were defined based on three cutoff values used to classify patients’ histologic activity with respect to neutrophils in the lamina propria (score ≥ 3.1 indicating active disease), neutrophils in both the lamina propria and in crypts (score ≥ 4.1 indicating active disease), and both crypt destruction and epithelium erosion (score ≥ 5.1 indicating active disease). Patients’ histologic score was also examined as a continuous outcome, with Geboes scores transformed to a continuous, ordinal scale, as has been recommended by Mosli et al. [47] and previously used by other researchers [48]. Transformed ordinal Geboes scores (TOGS) were based on only parameters 2B to 5, thus, excluding parameters related to chronic inflammation. When transforming to an ordinal score, a score increment of 1 point was assigned for all parameters starting with 2B upwards, with an additional point added for each subgrade (up to 3 subgrades for parameters 2B, 3, and 4, and 4 subgrades for parameter 5). The scoring key is presented in Table 3 in the ESM. Scores were then summed across the 4 parameters to result in the TOGS score, which ranged from 0 to 13 points. The TOGS score analyzed for each patient at each visit was the highest score among all biopsies taken for that patient at the visit, regardless of location.

#### Health-related quality of life

Patients’ HRQoL was assessed using both disease-specific and generic measures. The disease-specific measure was the Short Inflammatory Bowel Disease Questionnaire (SIBDQ) [49], which comprises the 10 items that best explained variability in scores from the 32 items on the original IBDQ questionnaire [50]. The SIBDQ assesses, over the previous 2-week period, the frequency and severity of the impact of patients’ UC on four domains of their health and functioning: bowel symptoms, systemic symptoms, emotional function, and social function. Patients’ generic HRQoL was measured using the 12-item SF-12v2^®^ Health Survey (SF-12v2) [51], which assesses 8 domains of patients’ functioning and well-being—physical functioning, role physical, bodily pain, general health, vitality, social functioning, role emotional, and mental health—over the previous 4-week period. More details of the domains assessed by the SIBDQ and SF-12v2 and scoring are provided in the Methods section in the ESM. Both patient-reported outcome measures were administered to patients electronically using IWRS.

### Statistical analysis

Descriptive statistics (means and standard deviations for continuous variables, and frequency and percentages for categorical variables) for patients’ demographic and clinical characteristics were calculated at baseline.

Associations between changes in SF-12v2 and SIBDQ domains and changes in disease activity (UC-DAI and TOGS histology scores) from baseline to end of treatment were examined using Spearman rank-order correlation coefficients. The magnitude of correlations was interpreted following Cohen’s guidelines (weak: *ρ* ≈ 0.1; moderate: *ρ* ≈ 0.3, strong: *ρ* ≈ 0.5) [52].

The degree to which HRQoL varied as a function of meaningful changes in disease activity and status was assessed by comparing SIBDQ and SF-12v2 domain scores between dichotomous patient subgroups. Patients were assigned to subgroups across four separate predetermined markers of disease improvement at the end of treatment: (1) achievement of clinical remission (scores of 0 on stool frequency and rectal bleeding UC-DAI items) versus non-achievement; (2) achievement of mucosal healing (a score of ≤ 1 on the mucosal appearance UC-DAI item) versus non-achievement; (3) improvement in stool frequency (a decrease of ≥ 1 point) versus no improvement; and (4) improvement in rectal bleeding (a decrease of ≥ 1 point) versus no improvement. Patients were also assigned to subgroups as a function of whether or not their maximum histology score at the week 8/EW exceeded each of the 3 cutoff values (3.1, 4.1, and 5.1). Scores on SIBDQ and SF-12v2 domains were compared between subgroups based on each of these 7 markers using independent-samples *t* tests. Hochberg’s method for adjusting *p* values [53] was applied across all pairwise comparisons within each marker to control for inflation of Type-I error due to multiplicity. The magnitude of subgroup differences in mean scores was evaluated by calculating Cohen’s *d* effect sizes for standardized mean differences and comparing them to Cohen’s interpretation guidelines (small effect: *d* ≈ 0.2; medium effect: *d* ≈ 0.5, large effect: *d* ≈ 0.8) [52].

The joint impact of clinical remission and mucosal healing status was evaluated by comparing whether HRQoL for patients achieving both was greater than for those achieving only one or neither. Because there were so few patients (*n* = 8) who did not achieve mucosal healing but did achieve clinical remission at week 8 (MH−/CR+), we determined that it was inappropriate to make statistical comparisons using this subgroup. Instead, we compared change in mean scores on SIBDQ and SF-12v2 domains among three subgroups—patients who achieved both mucosal healing and clinical remission (MH+/CR+), patients who achieved mucosal healing but not clinical remission (MH+/CR*−*), and patients who did not achieve either (MH−/CR−) at final visit—using univariate analysis of covariance (ANCOVA) models with baseline score as a covariate (with Hochberg-adjusted *p* values), and with planned pairwise comparisons to assess the marginal gain due for achieving clinical remission and/or mucosal health.

Multivariable linear regression models for change in each SIBDQ and SF-12v2 domain from baseline to final visit were conducted. Independent variables entered into each model included patients’ age, gender, and baseline body mass index (BMI), baseline score on the domain, and change from baseline to final visit for each of the 4 UC-DAI components and TOGS histology score. The statistical significance of variability in the outcome accounted for by each individual independent variable was assessed based on statistical tests for standardized regression weights.

All statistical models, which were post hoc for exploratory analyses following completion of the study, tested 2-tailed *p* values with *α* = 0.05. All statistical analyses were performed using SPSS for Windows, version 23 (2015; Armonk, NY: IBM Corp).

## Results

Descriptive statistics for patients’ baseline characteristics for the full induction phase efficacy population (*n* = 717) are presented in Table 1. All mean scores on UC-DAI components ranged between 1 and 2 points, indicating mild-to-moderate clinical and endoscopic symptoms. Mean SF-12v2 scores were below the general population average (i.e., < 50) on all domains, with deficits particularly large for domains capturing social functioning, role limitations, and bodily pain.

**Table 1:** Patient characteristics at baseline

Characteristic	Full sample (*N* = 717)
Female, *n* (%)	308 (43.0)
Age, mean (SD)	42.9 (14.0)
BMI, mean (SD)	24.4 (4.9)
UC-DAI, mean (SD)	
Stool frequency	1.7 (0.8)
Rectal bleeding severity	1.3 (0.7)
Mucosal appearance	1.9 (0.5)
Physician global assessment	1.6 (0.5)
Total score	6.6 (1.6)
Histology (modified Geboes scoring)	
Neutrophils in the lamina propria, *n* (%)^a^	590 (83.3)
Neutrophils in both the lamina propria and crypts, *n* (%)^b^	577 (81.5)
Crypt destruction and epithelium erosion, *n* (%)^c^	491 (69.4)
Histology (TOGS)	
TOGS score, mean (SD)	5.8 (3.9)
SIBDQ, mean (SD)	
Bowel symptoms	12.6 (3.1)
Systemic symptoms	8.9 (2.6)
Emotional function	12.8 (3.9)
Social function	8.9 (3.0)
SF-12v2, mean (SD)	
Physical functioning	46.3 (9.1)
Role physical	43.8 (7.9)
Bodily pain	43.5 (9.1)
General health	41.5 (10.1)
Vitality	46.8 (9.5)
Social functioning	42.4 (9.3)
Role emotional	41.9 (9.6)
Mental health	44.2 (9.4)

*SD* standard deviation, *BMI* body mass index, *UC-DAI* Ulcerative Colitis–Disease Activity Index, *SF-12v2* SF-12v2 Health Survey, *SIBDQ* Short Inflammatory Bowel Disease Questionnaire, *TOGS* transformed ordinal Geboes score

^a^Maximum histologic score ≥ 3.1

^b^Maximum histologic score ≥ 4.1

^c^Maximum histologic score ≥ 5.1

Correlations between UC-DAI component scores and SIBDQ domains (Table 2) were weak to moderate, ranging from − 0.18 (between mucosal appearance and systemic symptoms) to − 0.47 (between stool frequency and bowel symptoms). Correlations across all SIBDQ domains were generally largest for the stool frequency component and smallest for mucosal appearance. The SIBDQ bowel symptoms and emotional function domains showed the largest associations with UC-DAI scores, both with moderate correlations with component scores and strong correlations with UC-DAI total score, while the systemic symptoms domain was the least associated with UC-DAI scores. Correlations between UC-DAI component scores and SF-12v2 domains (Table 2) were also weak to moderate, ranging from − 0.16 (between mucosal appearance and vitality) to − 0.40 (between stool frequency and role physical). Following the same pattern observed for SIBDQ domains, the magnitudes of correlations across all SF-12v2 domains were generally largest for the stool frequency component and smallest for mucosal appearance. Moderate correlations with UC-DAI components were observed for bodily pain, social functioning, role physical, role emotional, and general health domains, while weak correlations were observed for mental health, vitality, and physical functioning domains. Changes in TOGS histology scores were weakly correlated with changes in all SIBDQ domains and all SF-12v2 domains.

**Table 2:** Spearman correlations between changes in UC-DAI and histology scores and changes in SIBDQ and SF-12v2 domain scores from baseline to the final visit of the 8-week induction period

	Total	UC-DAI component				Histology score
		Stool frequency	Rectal bleeding	Mucosal appearance	PGA	TOGS
SIBDQ domains						
Bowel symptoms	− 0.52	− 0.47	− 0.39	− 0.32	− 0.40	− 0.19
Systemic symptoms	− 0.33	− 0.30	− 0.26	− 0.18	− 0.26	− 0.05
Emotional function	− 0.47	− 0.38	− 0.34	− 0.32	− 0.37	− 0.14
Social function	− 0.52	− 0.45	− 0.36	− 0.33	− 0.39	− 0.14
SF-12v2 domains						
Physical functioning	− 0.32	− 0.26	− 0.24	− 0.24	− 0.27	− 0.07
Role physical	− 0.44	− 0.40	− 0.32	− 0.25	− 0.31	− 0.17
Bodily pain	− 0.41	− 0.39	− 0.30	− 0.25	− 0.32	− 0.12
General health	− 0.40	− 0.34	− 0.33	− 0.29	− 0.28	− 0.05
Vitality	− 0.35	− 0.35	− 0.29	− 0.16	− 0.24	− 0.12
Social functioning	− 0.43	− 0.37	− 0.31	− 0.28	− 0.33	− 0.12
Role emotional	− 0.40	− 0.37	− 0.26	− 0.25	− 0.32	− 0.09
Mental health	− 0.31	− 0.31	− 0.20	− 0.20	− 0.23	− 0.04

*UC-DAI* Ulcerative Colitis–Disease Activity Index, *SF-12v2* SF-12v2 Health Survey, *SIBDQ* Short Inflammatory Bowel Disease Questionnaire, *PGA* physician’s global assessment, *TOGS* transformed ordinal Geboes score

All SIBDQ and SF-12v2 domains were responsive to markers of improvement in disease activity, as indicated by statistically significant mean differences (all Hochberg-adjusted *p* < 0.001) between patients who achieved clinical or endoscopic remission or ≥ 1-point improvement in stool frequency or rectal bleeding from baseline to final study visit and those who did not (Table 3). Overall, SIBDQ domains were more responsive than SF-12v2 domains to all subgroup differences, with the distinction most prominent for achievement of mucosal healing, which had a large overall impact on SIBDQ domains (average *d* = 0.95) and a medium-sized impact across SF-12v2 scores (average *d* = 0.72). The SIBDQ bowel symptoms domain was consistently most responsive to the absence or presence in improvements in disease activity, while the systemic symptoms domain was consistently least responsive. Among SF-12v2 domains, general health, bodily pain, and social functioning were consistently most responsive to marker-based improvements in disease activity, while vitality, physical functioning, and mental health were consistently least responsive.

**Table 3:** Subgroup comparisons based on changes in UC-DAI components for mean changes in SIBDQ and SF-12v2 domain scores from baseline to the final visit of the 8-week induction period

Measure	Achieved clinical remission^a^			Achieved mucosal healing^b^			Improved stool frequency^c^			Improved rectal bleeding^d^		
	Yes (*n* = 292)	No (*n* = 394)	ES (*d*)	Yes (*n* = 542)	No (*n* = 117)	ES (*d*)	Yes (*n* = 431)	No (*n* = 251)	ES (*d*)	Yes (*n* = 445)	No (*n* = 240)	ES (*d*)
SIBDQ domains												
Bowel symptoms	5.7 (3.7)	3.2 (4.2)*	0.62	5.0 (3.8)	0.9 (4.1)*	1.06	5.5 (3.8)	2.3 (4.0)*	0.81	5.2 (3.9)	2.6 (4.1)*	0.65
Systemic symptoms	2.8 (2.7)	1.4 (2.8)*	0.49	2.4 (2.8)	0.4 (2.9)*	0.68	2.6 (2.9)	1.0 (2.6)*	0.55	2.5 (2.8)	1.1 (2.7)*	0.48
Emotional function	4.7 (4.3)	2.2 (4.0)*	0.61	4.1 (4.0)	0.0 (3.6)*	1.05	4.4 (4.3)	1.5 (3.7)*	0.69	4.2 (4.2)	1.7 (4.1)*	0.61
Social function	3.8 (3.3)	1.9 (3.4)*	0.56	3.3 (3.2)	0.2 (3.4)*	0.98	3.7 (3.4)	1.2 (3.0)*	0.77	3.5 (3.3)	1.4 (3.3)*	0.63
*Mean*			*0.57*			*0.95*			*0.71*			*0.59*
SF-12v2 domains												
Physical functioning	6.8 (10.8)	2.6 (10.1)*	0.40	5.8 (10.2)	– 0.9 (10.3)*	0.66	6.3 (10.9)	1.1 (9.3)*	0.51	6.3 (10.5)	0.9 (9.9)*	0.52
Role physical	7.9 (8.7)	4.0 (9.0)*	0.44	6.7 (8.7)	1.2 (9.4)*	0.63	8.1 (8.7)	1.5 (8.3)*	0.77	7.5 (8.8)	2.1 (8.6)*	0.62
Bodily pain	11.0 (9.6)	5.8 (10.9)*	0.50	9.9 (9.5)	1.4 (12.0)*	0.85	10.7 (10.0)	3.6 (10.5)*	0.70	10.0 (10.1)	4.5 (10.9)*	0.52
General health	10.8 (10.5)	4.8 (11.2)*	0.55	9.3 (10.2)	0.1 (11.9)*	0.87	10.2 (10.9)	2.5 (10.3)*	0.72	9.8 (10.9)	2.9 (10.7)*	0.64
Vitality	8.6 (11.4)	4.5 (11.5)*	0.36	7.5 (11.4)	1.7 (11.1)*	0.51	8.9 (11.3)	1.7 (10.8)*	0.64	8.3 (11.6)	2.6 (10.8)*	0.50
Social functioning	9.5 (10.1)	4.8 (10.8)*	0.45	8.5 (10.0)	0.3 (10.8)*	0.81	9.2 (10.4)	2.6 (10.2)*	0.64	8.9 (10.3)	2.7 (10.4)*	0.60
Role emotional	8.6 (10.5)	3.3 (10.7)*	0.50	7.2 (10.2)	– 0.2 (11.2)*	0.71	8.3 (10.3)	0.8 (10.5)*	0.72	7.3 (10.7)	2.2 (10.7)*	0.48
Mental health	9.3 (11.5)	4.4 (10.7)*	0.44	8.1 (10.7)	0.6 (11.4)*	0.69	8.8 (11.4)	2.5 (9.8)*	0.58	8.2 (11.3)	3.4 (10.6)*	0.43
*Mean *			*0.46*			*0.72*			*0.66*			*0.54*

Data are means (standard deviations)

*UC-DAI* Ulcerative Colitis–Disease Activity Index, *SF-12v2* SF-12v2 Health Survey, *SIBDQ* Short Inflammatory Bowel Disease Questionnaire, *ES* effect size

*Hochberg-adjusted *p* < 0.001

^a^Clinical remission defined as scores of 0 on both stool frequency and rectal bleeding items of the UC-DAI at the final visit

^b^Mucosal healing defined as a score of 0 or 1 on the mucosal appearance item of the UC-DAI at the final visit

^c^Improvement in stool frequency was defined as a decrease of ≥ 1 point on the stool frequency item of the UC-DAI at the final visit

^d^Improvement in rectal bleeding was defined as a decrease of ≥ 1 point on the rectal bleeding item of the UC-DAI at the final visit

Scores on SIBDQ and SF-12v2 domains at patients’ final visit varied as a function of their histologic disease activity at that visit, as indicated by statistically significant mean differences (all Hochberg-adjusted *p* < 0.05) between subgroups of patients classified by the cutoff values of 3.1, 4.1, and 5.1 (Table 4). The largest differences in SIBDQ and SF-12v2 mean scores were observed between subgroups based on the highest cutoff value (5.1 points); differences in SIBDQ and SF-12v2 domains were generally comparable between subgroups defined by cutoff values of 3.1 and 4.1. Consistent with findings across clinical and endoscopic subgroups, SIBDQ domains were generally more responsive to patients’ histologic disease status than SF-12v2 domains. Again, the SIBDQ systemic symptoms domain was consistently least responsive to disease activity; responsiveness was similar among the other 3 SIBDQ domains. Among SF-12v2 domains, social functioning was consistently most responsive to histologic disease activity, with general health consistently the least responsive.

**Table 4:** Subgroup comparisons based on maximum histology cutoff values for mean SIBDQ and SF-12v2 domain scores at the final visit of the 8-week induction period

Measure	Neutrophils in the lamina propria^a^			Neutrophils in both the lamina propria and crypts^b^			Crypt destruction and epithelium erosion^c^		
	No (*n* = 296)	Yes (*n* = 371)	ES (*d*)	No (*n* = 311)	Yes (*n* = 356)	ES (*d*)	No *(n* = 403)	Yes (*n* = 264)	ES (*d*)
SIBDQ domains									
Bowel symptoms	17.6 (2.8)	16.3 (3.8)***	0.39	17.6 (2.8)	16.2 (3.8)***	0.41	17.6 (2.8)	15.8 (4.0)***	0.52
Systemic symptoms	11.3 (2.3)	10.8 (2.5)*	0.22	11.3 (2.2)	10.7 (2.6)*	0.25	11.2 (2.3)	10.6 (2.6)**	0.26
Emotional function	17.0 (3.3)	15.5 (4.3)***	0.39	17.0 (3.2)	15.4 (4.3)***	0.41	16.9 (3.2)	15.0 (4.5)***	0.51
Social function	12.4 (2.1)	11.2 (3.1)***	0.44	12.4 (2.1)	11.2 (3.1)***	0.45	12.3 (2.2)	10.9 (3.2)***	0.55
*Mean*			*0.36*			*0.38*			*0.46*
SF-12v2 domains									
Physical functioning	52.4 (7.2)	50.4 (8.7)*	0.24	52.2 (7.3)	50.5 (8.7)*	0.22	52.3 (7.3)	49.7 (9.1)***	0.31
Role physical	50.7 (6.5)	49.0 (7.9)*	0.23	50.7 (6.4)	48.9 (8.0)*	0.25	50.9 (6.4)	47.8 (8.4)***	0.43
Bodily pain	53.3 (6.5)	50.9 (8.9)**	0.30	53.3 (6.4)	50.8 (8.9)**	0.31	53.1 (6.6)	50.1 (9.5)***	0.38
General health	50.0 (8.3)	48.3 (9.9)*	0.18	50.0 (8.3)	48.3 (9.9)*	0.18	50.0 (8.6)	47.6 (10.1)**	0.26
Vitality	55.1 (8.5)	52.4 (10.0)**	0.29	55.0 (8.5)	52.4 (10.1)**	0.29	54.9 (8.7)	51.5 (10.3)***	0.36
Social functioning	51.2 (7.3)	48.2 (9.2)***	0.36	51.3 (7.2)	48.1 (9.3)***	0.38	51.2 (7.4)	47.0 (9.6)***	0.51
Role emotional	48.9 (8.2)	47.1 (9.4)*	0.20	48.9 (8.1)	47.0 (9.6)*	0.21	49.0 (8.0)	46.1 (10.0)***	0.33
Mental health	52.4 (8.5)	50.0 (10.0)*	0.25	52.5 (8.4)	49.8 (10.1)**	0.29	52.3 (8.3)	49.1 (10.6)***	0.34
*Mean *			*0.26*			*0.27*			*0.37*

*SIBDQ* Short Inflammatory Bowel Disease Questionnaire,* SF-12v2* SF-12v2 Health Survey,*ES* effect size

*Hochberg-adjusted *p* < 0.05

**Hochberg-adjusted *p* < 0.01

***Hochberg-adjusted *p* < 0.001

^a^Maximum histology score ≥ 3.1 at final visit

^b^Maximum histology score ≥ 4.1 at final visit

^c^Maximum histology score ≥ 5.1 at final visit

**Fig. 1:**
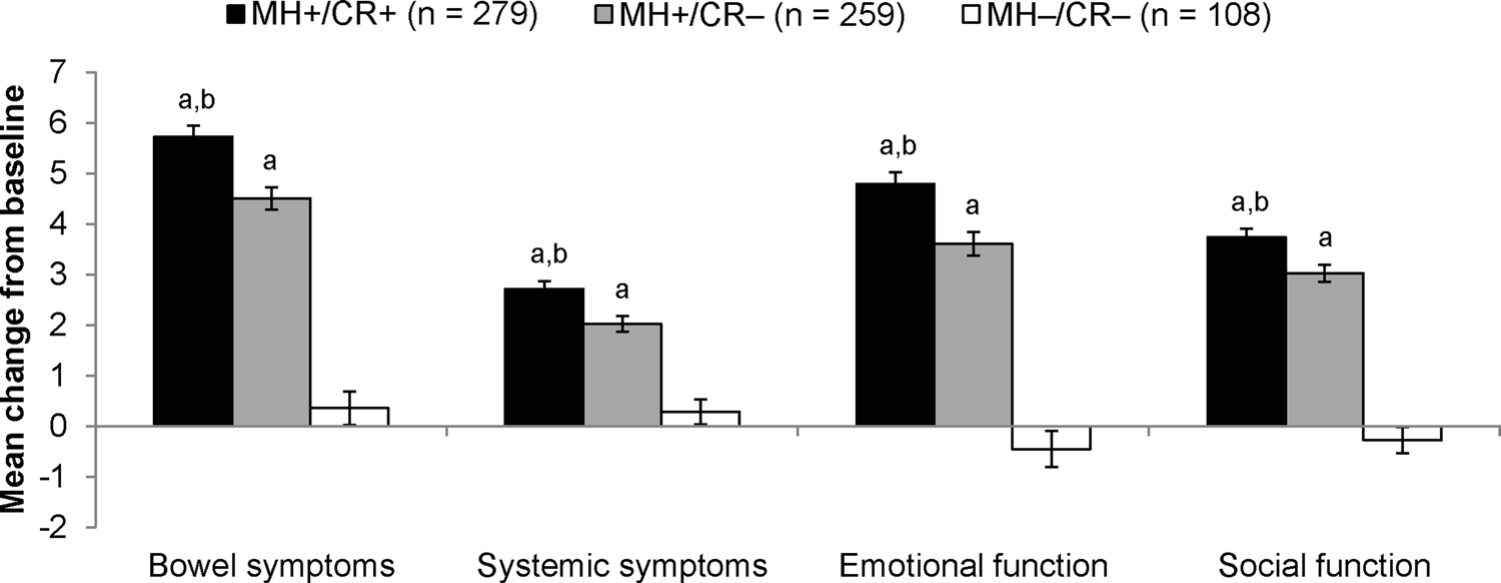
Change in mean SIBDQ domain scores from baseline to the final visit of the 8-week induction period for patients who achieved mucosal healing with or without clinical remission. *MH*+ achieved mucosal healing, *CR*+ achieved clinical remission, *CR−* did not achieve clinical remission, *MH−* did not achieve mucosal healing. The MH−/CR+ subgroup was not included in the analysis because it included only eight patients. Error bars represent standard errors of means. ^a^Improvement is statistically significantly larger than for MH−/CR*−* (Hochberg-adjusted *p* < 0.001). ^b^Improvement is statistically significantly larger than for MH+/CR*−* (Hochberg-adjusted *p* < 0.01)

**Fig. 2:**
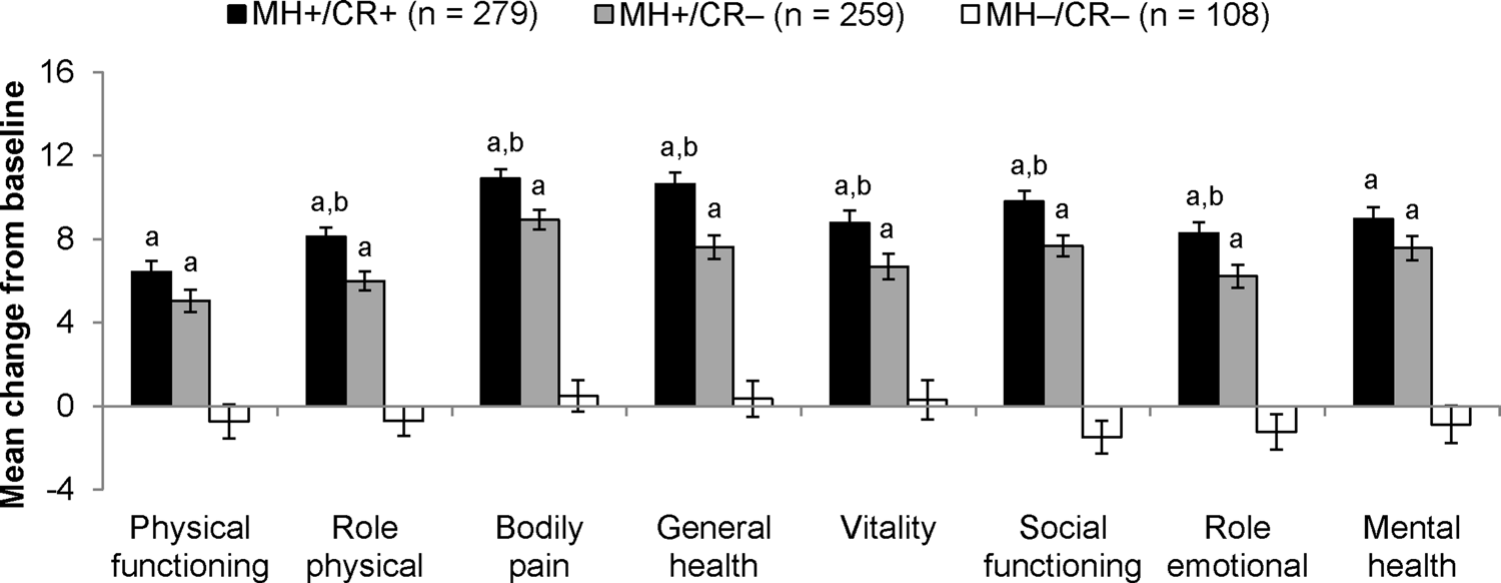
Change in mean SF-12v2 domain scores from baseline to the final visit of the 8-week induction period for patients who achieved mucosal healing with or without clinical remission. *MH*+ achieved mucosal healing, *CR*+ achieved clinical remission, *CR−* did not achieve clinical remission, *MH−* did not achieve mucosal healing. The MH−/CR+ subgroup was not included in the analysis because it included only eight patients. Error bars represent standard errors of means. ^a^Improvement is statistically significantly larger than for MH−/CR*−* (Hochberg-adjusted *p* < 0.001). ^b^Improvement is statistically significantly larger than for MH+/CR*−* (Hochberg-adjusted *p* < 0.05)

Comparisons of mean change in scores from baseline to final visit among MH+/CR+, MH+/CR−, and MH−/CR− subgroups are presented in Fig. 1 for SIBDQ domains and Fig. 2 for SF-12v2 domains. For all domains of both instruments, patients who achieved both mucosal healing and clinical remission or mucosal healing alone showed statistically larger improvements than patients who achieved neither mucosal healing nor clinical remission (all Hochberg-adjusted *p* < 0.001). Further, for all domains of the SIBDQ and for 6 of the 8 SF-12v2 domains (all but physical functioning and mental health), patients who achieved both mucosal healing and clinical remission scored better than patients who achieved mucosal healing but not clinical remission (all Hochberg-adjusted *p* < 0.05).

Findings from multivariable linear regression models are presented in Table 5. Patients’ baseline characteristics (age, gender, and BMI) were not significant predictors of changes for the majority of SIBDQ and SF-12v2 domains. Baseline values for each domain were strong and statistically significant (all *p* < 0.001) predictors of decreased scores, such that patients with worse values at baseline showed greater improvements following treatment. Each of the 4 UC-DAI components accounted for a statistically significant amount of unique variance for the majority of HRQoL domains. For the SIBDQ, all 4 components were significantly associated with bowel symptoms and emotional and social function domain scores, and all but mucosal appearance and physician’s global assessment were significantly associated with change in scores on the systemic symptoms domain. Across SIBDQ domains, regression weights for stool frequency (*β*s ranging from − 0.113 to − 0.187) and physician’s global assessment (*β*s ranging from − 0.087 to − 0.181) were generally numerically larger than for mucosal appearance and rectal bleeding. Across UC-DAI components, regression weights were generally numerically largest for changes in bowel symptoms (*β*s ranging from − 0.109 to − 0.187) and generally numerically smallest for changes in systemic symptoms (*β*s ranging from − 0.038 to − 0.113). For the SF-12v2, changes in stool frequency were associated with statistically significant changes in all domains, while changes in each of the other 3 components were associated with significant changes for at least half of the domains (role physical, bodily pain, general health, and vitality for rectal bleeding; role physical, bodily pain, general health, social functioning, and role emotional for mucosal appearance; and role physical, bodily pain, social functioning, role emotional, and mental health for physician’s global assessment). Across SF-12v2 domains, regression weights for stool frequency (*β*s ranging from − 0.083 to − 0.177) were generally numerically larger than for the other UC-DAI components (*β*s ranging from − 0.044 to − 0.164). Across UC-DAI components, regression weights were generally numerically smallest for changes in physical functioning, vitality, and mental health domains (*β*s ranging from − 0.047 to − 0.120). Change in patients’ TOGS histology score did not account for significant variability in any SIBDQ or SF-12v2 domains.

**Table 5:** Change in SIBDQ and SF-12v2 domain scores from baseline to the final visit of the 8-week induction period in multivariable linear regression models

Outcome	Standardized regression weights									Adjusted * R*^2^
	Baseline characteristics				Disease activity					
					UC-DAI components				Histology	
	Age	Sex	BMI	Baseline value of outcome variable	Stool frequency	Rectal bleeding	Mucosal appearance	PGA	TOGS	
SIBDQ										
Bowel symptoms	0.034	0.008	0.007	− 0.554***	− 0.187***	− 0.109**	− 0.133**	− 0.181***	− 0.023	0.555
Systemic symptoms	0.054	0.012	− 0.048	− 0.592***	− 0.113**	− 0.099*	− 0.038	− 0.087	− 0.016	0.435
Emotional function	0.036	0.096**	− 0.024	− 0.526***	− 0.121**	− 0.111**	− 0.113*	− 0.152**	− 0.044	0.472
Social function	− 0.028	0.061	0.021	− 0.624***	− 0.140***	− 0.077*	− 0.166***	− 0.114**	− 0.020	0.591
SF-12v2										
Physical functioning	− 0.043	− 0.013	− 0.009	− 0.654***	− 0.083*	− 0.048	− 0.064	− 0.068	− 0.039	0.482
Role physical	0.003	− 0.005	− 0.023	− 0.618***	− 0.177***	− 0.079*	− 0.084*	− 0.107**	− 0.045	0.537
Bodily pain	− 0.031	0.018	− 0.026	− 0.673***	− 0.131***	− 0.114***	− 0.091*	− 0.080*	− 0.023	0.618
General health	− 0.046	0.005	− 0.028	− 0.591***	− 0.152***	− 0.164***	− 0.125**	− 0.044	0.049	0.510
Vitality	0.015	− 0.041	− 0.086*	− 0.595***	− 0.120**	− 0.109**	− 0.049	− 0.084	0.016	0.473
Social functioning	− 0.014	0.021	− 0.034	− 0.623***	− 0.125***	− 0.064	− 0.126**	− 0.095*	− 0.052	0.536
Role emotional	− 0.023	0.006	− 0.033	− 0.611***	− 0.151***	− 0.048	− 0.090*	− 0.087*	− 0.037	0.488
Mental health	0.054	− 0.003	− 0.119**	− 0.577***	− 0.099*	− 0.047	− 0.084	− 0.100*	− 0.007	0.424

*UC-DAI* Ulcerative Colitis–Disease Activity Index, *SF-12v2* SF-12v2 Health Survey, *SIBDQ* Short Inflammatory Bowel Disease Questionnaire, *PGA* physician’s global assessment, *TOGS* transformed ordinal Geboes score, *BMI* body mass index

*p < 0.05

**p < 0.01

***p < 0.001

## Discussion

Researchers have recently suggested that assessment of disease activity of patients with UC by clinicians should incorporate evaluations of 4 factors: clinical symptoms, endoscopy, histology, and HRQoL [54].

Historically, in the 1980s and 1990s, treatment benefit was defined as improvement or remission in clinical symptoms [55, 56]. During the first decade of the 2000s, expert consensus was reached that classification of treatment response or remission, both in clinical trials and in practice, needed to also include direct evidence of mucosal healing as measured by endoscopy [14, 56–58]. The importance of including mucosal healing when assessing patients’ disease activity has been indirectly supported by evidence that induction of clinical and endoscopic remission is strongly associated with improvements in HRQoL [31, 33, 36, 59]. Within the past decade, many researchers have claimed that establishing “complete” or “deep” remission in patients with UC requires histologic remission in addition to clinical and endoscopic remission, and that this should be a target therapeutic goal [26–29]. However, to this point, the contribution of histologic healing and HRQoL has not been thoroughly examined in patients with UC.

Findings from the current analyses support previous evidence that both improved clinical symptoms and mucosal healing were associated with improvements in HRQoL for patients with active mild-to-moderate UC. Changes in scores on each component of the UC-DAI from baseline to patients’ final visit were moderately correlated with changes in scores on most SIBDQ and SF-12v2 domains, particularly bowel symptoms, emotional function, and social function domains of the former, and role–physical, bodily pain, general health, social functioning, and role–emotional domains of the latter. Patients who displayed meaningful improvements in key clinical and endoscopic activity markers, such as achievement of clinical or endoscopic remission, had substantially larger improvements in SIBDQ and SF-12v2 domain scores than those who did not, as did patients who achieved both endoscopic and clinical remission compared to patients who achieved endoscopic remission only. In addition, patients with histology indicating active disease at the end of treatment scored significantly worse on all SIBDQ and SF-12v2 domains than patients with no inflammation. Finally, decreases in each of the UC-DAI component scores, even when controlling for variance shared with the other three UC-DAI components, were statistically significantly correlated with improvement in scores for the majority of SIBDQ and SF-12v2 domains, though change in histology (TOGS) was not correlated with changes in HRQoL outcomes.

Patients who achieved clinical remission, who demonstrated full mucosal healing, or who showed meaningful reductions in stool frequency or rectal bleeding reported significantly larger improvements in all HRQoL domains than did their counterparts. However, among each of these factors, achievement of mucosal healing produced the largest subgroup differences for HRQoL improvements. The driving factor behind this larger difference was not that achieving mucosal healing led to greater improvement than did meeting other markers: across all markers, the mean change in domain scores were relatively comparable for those showing improved disease status. Rather, the distinction of mucosal healing from the other factors is most apparent when examining those who failed to demonstrate improvement. Patients who did not achieve clinical remission still demonstrated some improvement in HRQoL, with increases on SIBDQ domains ranging from 1.4 points (systemic symptoms) to 3.2 points (bowel symptoms) and on SF-12v2 domains from 2.6 points (physical functioning) to 5.8 points (bodily pain). In contrast, patients who did not exhibit endoscopic remission at their final visit showed on average only trivial improvements in HRQoL, with increases < 1 point on all SIBDQ domains and < 2 points on all SF-12v2 domains. Taken together, these findings indicate that mucosal healing, unlike symptom reduction, may be a necessary condition for improvement of HRQoL for patients with UC.

Results from regression models provide further support for the independent contribution of clinical symptoms and mucosal health to HRQoL. Each component of the UC-DAI was uniquely associated with significant variability in the majority of SIBDQ and SF-12v2 domains, indicating that clinical and endoscopic activity of patients with UC each have at least some distinct, and thus additive, associations with their HRQoL.

Results from both correlation and regression models found a lack of linear relationships between changes from baseline to final visit in histology score (TOGS) and changes in SIBDQ or SF-12v2 domains. However, post-treatment comparisons of HRQoL across groups classified by predefined markers of histologic disease activity indicated that histologic status was statistically associated with patients’ concurrent HRQoL. Because these two analyses differed in both how histology was scaled (as a continuous or categorical variable) and whether using current histology status or change in histology status, a post hoc analysis calculated Spearman correlations between TOGS at the final visit with concurrent HRQoL scores. Magnitudes of all correlations were very small, suggesting that differences in HRQoL as a function of histologic disease activity may vary as a function of kind, as defined by clinically meaningful categories, rather than degree.

Across all analyses, some consistent patterns emerged with respect to the relative strength of associations among specific dimensions of disease activity with HRQoL. One such pattern of findings pointed to changes in stool frequency being more related to changes in HRQoL than were changes in rectal bleeding. Median correlations across domains were slightly larger for changes in stool frequency than rectal bleeding for both the SIBDQ and SF-12v2. Differences in HRQoL for patients achieving improvement as compared to those who did not were also larger for stool frequency than rectal bleeding, with average effect sizes across SIBDQ and SF-12v2 domains being larger for the comparison of stool frequency subgroups than for the comparison of rectal bleeding subgroups. Finally, in all but one of the multivariable regression models (the exception being the model for the general health domain of the SF-12v2), the standardized regression weight for change in stool frequency was of greater magnitude than that for rectal bleeding.

The analyses reported here included data from assessments for only the induction phase of the trial, and not for the maintenance phase. The rationale for this decision was related to the established finding that detecting associations among changes in outcomes, which is the focus here, is impacted by the amount of variability in these changes, such that a restricted range of values among variables attenuates their intercorrelations [60]. As would be expected, during the induction phase, when patients with active UC (and thus poor clinical and HRQoL outcomes) received treatment, the majority of patients showed improvement in these outcomes, with large variation in the magnitudes of change across patients [61]. However, also as would be expected, in the maintenance phase, which enrolled only patients in partial or full remission who then continued to receive treatment, changes in clinical and HRQoL outcomes were observed for a minority of patients, with little variation in the magnitudes of change across patients [44, 61]. This restricted range of values in the maintenance phase would likely underestimate the associations among changes in clinical and HRQoL outcomes. Thus, it was determined that the objectives of the current analyses would be best served by including data from only the induction phase of the MOMENTUM trial.

One limitation of the current clinical trial is the use of shortened instruments for assessing patients’ disease activity and HRQoL, which restricts the precision of measurement for each of these outcomes. While the UC-DAI and similar indices capturing both clinical and endoscopic activity in patients with UC (e.g., Mayo score [62]) are frequently included as efficacy endpoints in clinical trials, they are by design simplified measures of these outcomes. In particular, the UC-DAI is limited both in the scope of health outcomes measured—only two clinical symptoms, and no assessment of histologic or biochemical disease activity—and in the variability of possible outcomes due to all items being scored on a highly compressed 4-point scale. An instrument that captures additional clinical symptoms and uses an expanded scale to capture finer distinctions in disease activity could improve the breadth and precision of estimates of patients’ disease activity, leading to more accurate assessments about their association with other variables, such as HRQoL. Similarly, the SIBDQ and SF-12v2 are, by design, simplified measures: each was developed as a subset of items from a more comprehensive scale, specifically the IBDQ and the SF-36v2^®^ Health Survey (SF-36v2). While previous studies of patients with UC have provided evidence supporting the sensitivity to change and construct validity in UC samples of the SIBDQ [13, 49, 63, 64] and SF-12v2 [10, 32], using their parent instruments would provide more precise estimates of patients’ HRQoL. Further, while responder definitions, or thresholds indicating clinically meaningful change, have been established for scores on both the IBDQ and the SF-36v2 [65, 66], they have not been established for domain scores of the SIBDQ or SF-12v2. Thus, we cannot make inferences from these results as to whether the magnitudes of changes observed are clinically meaningful or relevant.

Another limitation of this study is the inability to examine how disease activity and HRQoL covary over time during treatment. It is possible that the relative magnitudes of associations between clinical symptoms or mucosal health and HRQoL (or, perhaps, with specific aspects of HRQoL) change over time. For example, it may be that clinical symptom reduction has a strong impact on HRQoL early in treatment, while the impact of mucosal healing of HRQoL appears later in the course of treatment. However, due to the lack of interim assessments of HRQoL during the induction phase of the MOMENTUM trial, changes in associations across different durations could not be assessed. While it may appear that including HRQoL data from patients during the maintenance phase of this trial would allow for comparing the magnitudes of associations at multiple assessments, this would in fact not be the case, for two reasons. First, patients who entered the maintenance phase were only a subset of patients who completed the induction phase—specifically, those who achieved partial or complete remission. Thus, it would not be possible to compare associations at week 8 of the induction phase with those at the end of the maintenance phase, as the patient samples for each of the two phases were not the same. Second, the maintenance phase, while much longer than the induction phase (12 months vs. 8 weeks), also did not include interim assessments of HRQoL. Thus, trials incorporating multiple assessments of these variables would be needed to provide important information about the development of these relations over the time course of the disease and its treatment.

The inclusion of only patients with mild-to-moderate UC in this study sample limits the ability to generalize the current findings to the full patient population, which also includes patients with more severe disease. Future research that examines the association between clinical, endoscopic, and histologic activity with HRQoL for patients with severe UC would be needed to understand whether the current findings are applicable to patients with UC across the entire range of disease severity.

Despite these limitations, results from this study, which is the first to simultaneously examine the individual impact of clinical, endoscopic, and histologic activity on HRQoL for patients with UC, provide clear evidence that clinical symptoms and mucosal health have separable, distinct, additive impacts on HRQoL of patients with UC. Treatments that target both clinical and mucosal health will likely result in greater improvements in patients’ HRQoL than those that are directed at only one or the other. Thus, these findings are supportive of the recently recognized importance of using evidence for “complete” remission when evaluating the effectiveness of treatment in clinical trials and hopefully to some extent in clinical practice. Achieving “complete” remission of UC, which includes histologic remission in addition to both clinical remission and mucosal healing, contributes to the ultimate therapeutic goal of treatment for patients with UC, which is to improve HRQoL [27].

## Supplementary Information

Below is the link to the electronic supplementary material.

Electronic supplementary material 1 (PDF 222 kb)

## Footnotes

1 The modification involved a change in classification on the endoscopic item, such that evidence of friability was coded as an indicator of ‘moderate disease,’ rather than as ‘mild disease’ as in the original version of the UC-DAI [42].

2 The modification involved a change in numbering, such that a score of 2A was recoded as a score of 2; 2B was recoded to 3; 3 was recoded to 4; 4 was recoded to 5; and 5 was recoded to 6.
