# Supplementary material for: The impact of clinical symptoms and endoscopic and histologic disease activity on health-related quality of life in patients with ulcerative colitis following treatment with multimatrix mesalazine
Source: Qual Life Res. 2021 Mar 2;30(7):1925–38. doi: 10.1007/s11136-021-02787-4 (PMC8233235; doi:10.1007/s11136-021-02787-4)
Supplement: Supplementary file 1 — Electronic supplementary material 1 (PDF 222 kb) [file 11136_2021_2787_MOESM1_ESM.pdf]

Online Resource

Quality of Life Research

The Impact of Clinical Symptoms and Endoscopic and Histologic Disease Activity on Health-related Quality of Life in Patients with Ulcerative Colitis Following Treatment with Multimatrix Mesalazine

Aaron Yarlas, PhD*, Mary Kaye Willian, DrPH, Arpita Nag, PhD

*QualityMetric, Johnston, RI, USA; Email: ayarlas@qualitymetric.com.

Supplemental Methods

Assessments

Health-related quality of life

Patients' HRQoL was assessed using both a disease-specific measure and a generic measure. The disease-specific measure was the Short Inflammatory Bowel Disease Questionnaire (SIBDQ) [45]. The SIBDQ comprises the 10 items that best explained variability in scores from the 32 items on the original IBDQ questionnaire [46]. The SIBDQ assesses, over the previous 2-week period, the frequency and severity of the impact of patients' UC on 4 domains of their health and functioning: bowel symptoms (problems with passing gas, abdominal pain, and stool urgency), systemic symptoms (fatigue and problems maintaining weight), emotional function (feelings of depression, tension, and anger), and social function (interference with social engagements and leisure activities). Each item is scored on a 7-point Likert-type scale, with values of 1 to 7. Domains are calculated as the sum of item scores, while a total score (range: 0 to 70) is calculated as the sum of all 10 items. For all domain scores and total score, higher scores reflect better quality of life. The SIBDQ has demonstrated good psychometric properties when used with UC patients [45, 47].

Patients' generic HRQoL was measured using the SF-12v2® Health Survey (SF-12v2) [48]. The 12 items of the SF-12v2, which has a 4-week recall period, assess 8 domains of patients' functioning and well-being: physical

functioning (PF), role limitations due to physical health problems (RP), bodily pain (BP), perception of general health (GH), vitality (VT), social functioning (SF), role limitations due to emotional health problems (RE), and mental health (MH). Each domain score is standardized into a *T*-score, with a mean of 50 and a standard deviation (SD) of 10 [48]. Weighted combinations of domain scores are used to derive *T*-scores for summaries of physical well-being (physical component score [PCS]) and mental well-being (mental component score [MCS]). For all domain scores and summary scores, higher scores reflect better quality of life.

Supplemental Table 1. List of Independent Ethics Committees or Institutions Review Boards.

IEC or IRB	Name of Committee Chair
Imelda vzw Commissie Medische Ethiek	Marc Lambrechts
Central IRB Services	Jean Mitchell
Multicentric Ethics Committee FN Hradec Kralove	Jiri Vortel
Comité de Protection des Personnes Sud-Est VI	Jean-Etiene BAZIN, Préside
Medical Faculty Ethics Committee	Wolfgang Poser
MRC-ECCP	Zsuzsanna Fürst
Beaumont Research Ethics Committee	Gillian Vale
NEC Comisia Nationala de Etica	Sava Dumitrescu
Ethics Council of Ministry of H/care & Social Dev	M.A. Sheveleva
CEIC Hospital Universitario La Princesa	Cecilia López
National Research Ethics Service	Martin Hewitt
Nottingham Research Ethics Committee 2	Linda Rose
Schulman Associates IRB	Laura Hall
Central IRB Services	Jean Mitchell
Medische Ethische CommissieH.-Hartziekenhuis Roese	L. Marcelis
Ethisch Comité AZ Groeninge	Lysebeth Luc
Comitê de Ética em Pesquisa em Seres Humanos Do HC	Renato Tambara Filho
CE em Pesquisa Clinica do Hospital Mae de Deus PoA	
Comité d'Ethque Hospital St-Sacrement	Ana Marin
Faculty of Health Sciences Research Ethics Board	Suzette Slama
Comité de Ética en Investigación Clínica Las Ameri.	Rodrigo Diaz Calle
Comité de Ética en Investigación en el área de la.	Gloria Visbal Illera
Comité de Investigaciones y Ética en Investigacion	Tatiana Lopez
Comité de Ética de la Investigación Riesgo de Frac	Nelson F. Castañeda Alarco
Etická Komise Nemocnice Tábor, a.s	Kamil Klenha
Etická Komise pri Orlickoústecké Nemocnici, a.s.	Miroslav Kares
Etická Komise Klinického Centra ISCARE	Vaclava Vodickova
Etická Komise Nemocnice Jablonec nad Nisou	Ota Feldman
Etická Komise – Krajská Zdravotní, a.s. - Masaryk	Jirí Král
Etická Komise IKEM a FTNsP	Vladimir Stanek
Lokální Etická Komise FN a LF UK v Plzni	Otto Mayer
Etická Komise Nemocnice Ceské Budejovice	Frantisek Vorel
Ethik-Kommission der Georg-August Universität Gött	W. Poser
Ethikkommission der MHH	W. Poser
Central India Medical Research Ethics Committee	S.M. Patil
Ethics Committee	D. Bhagabati
S.K. Kalla Memorial Ethical Committee for Human Res	B.K. Tiwari
Ethics Committee	B.G. Sahasrabuddhe
Independent Ethics Committee	Bupendra Shah
Institutional Ethics Committee	K. Surya Rao
Sanjeevani Ethics Committee	Ramesh Roop Rai
Ethics Committee	C.S. Bhaskaran

IEC or IRB	Name of Committee Chair
Institutional Ethics Committee (DTEC)	Tej K. Kaul
Ethics Committee of Poona Hospital & Research Cent.	Neena Luthra
Institutional Ethics Committee	Padmaja Udayakumar
Institutional Ethics Committee	U.C. Chaturvedi
SREE Gokulam Medical College and Research Found.	Sudheendra Ghosh
Komisja Bioetyczna przy Okregowej Izbie Lekarskiej	Jerzy Umiastowski
Pharma Ethics (Pty) Ltd	Marzelle Haskins
EC Hospital Universitario Dr. Peset	Genoveva Pallares Sancho-Tello
EC Hospital Universitario Ramón y Cajal	Yolanda Solsano
The VA Long Beach Healthcare System IRB	Kathleen Herron
Institutional Review Board – University of Chicago	Christopher Daugherty
Mayo Clinic Institutional Review Board	Joseph K. Lobl

IEC, Independent Ethics Committee; IRB, Institutional Review Board.

Supplemental Table 2. Coding for component scores of the modified Ulcerative Colitis Disease Activity Index (UC-DAI) administered in the MOMENTUM trial.

**Description**	**Component score**
**Stool frequency**	
Normal	0
1-2 more than normal/day	1
3-4 more than normal/day	2
>4 more than normal/day	3
**Rectal bleeding (most severe bleeding of the day)**	
None	0
Streaks of blood	1
Obvious blood	2
Mostly blood	3
**Mucosal appearance (endoscopy score)**	
Normal (intact vascular pattern, no friability or granulation)	0
Mild (erythema, decreased vascular pattern, minimal granularity)	1
Moderate (marked erythema, granularity, friability, absent vascular pattern, bleeding with minimal trauma, no ulcerations)	2
Severe (ulceration, spontaneous bleeding)	3
**Physician's Global Assessment**	
No active disease	0
Mild disease	1
Moderate disease	2
Severe disease	3

Supplemental Table 3. Scoring key for transformed ordinal Geboes score (TOGS).

Grade	Description	Ordinal score
	**Structural (architectural change)**	
0	No abnormality	0
0.1	Mild abnormality	0
0.2	Mild or moderate diffuse or multifocal abnormalities	0
0.3	Severe diffuse or multifocal abnormalities	0
	**Chronic inflammatory infiltrate**	
1	No increase	0
1.1	Mild but unequivocal increase	0
1.2	Moderate increase	0
1.3	Marked increase	0
	**Lamina propria eosinophils**	
2A.0	No increase	0
2A.1	Mild but unequivocal increase	0
2A.2	Moderate increase	0
2A.3	Marked increase	0
	**Lamina propria neutrophils**	
2B.0	No increase	0
2B.1	Mild but unequivocal increase	1
2B.2	Moderate increase	2
2B.3	Marked increase	3
	**Neutrophils in epithelium**	
3	None	0
3.1	<5% crypt involved	1
3.2	<50% crypt involved	2
3.3	>50% crypt involved	3
	**Crypt destruction**	
4	None	0
4.1	Probable-local excess of neutrophils in part of crypt	1
4.2	Probable-marked attenuation	2
4.3	Unequivocal crypt destruction	3
	**Erosion or ulceration**	
5	No erosion, ulceration or granulation tissue	0
5.1	Recovering epithelium plus adjacent inflammation	1
5.2	Probable erosion-focally stripped	2
5.3	Unequivocal erosion	3
5.4	Ulcer or granulation tissue	4
